# Preoperative Virtual Reality for Pediatric Patients Undergoing General Anesthesia: A Meta‐Analysis of Randomized Controlled Trial

**DOI:** 10.1111/pan.70016

**Published:** 2025-07-23

**Authors:** Difang Zhao, Ting Tian, Shuguang Jin

**Affiliations:** ^1^ Operating Room of Anesthesia Surgery Center West China Hospital Chengdu China; ^2^ West China School of Nursing Sichuan University Chengdu China; ^3^ Department of Pediatric Surgery West China Hospital Chengdu China

**Keywords:** general anesthesia, meta‐analysis, pediatric patients, perioperative outcomes, virtual reality

## Abstract

**Background:**

Pediatric patients undergoing general anesthesia often face stress responses, anesthetic challenges, and delayed recovery. Virtual reality has emerged as a promising non‐pharmacological intervention, though its effectiveness varies across studies. This meta‐analysis evaluates the effects of preoperative virtual reality interventions on various outcomes in pediatric patients.

**Methods:**

PubMed, EMBASE, the Cochrane Library, and Web of Science were searched for randomized controlled trials with intention‐to‐treat analysis comparing preoperative virtual reality interventions with standard care in pediatric patients undergoing general anesthesia. Primary outcomes included preoperative anxiety (Modified Yale Preoperative Anxiety Scale, change in Modified Yale Preoperative Anxiety Scale) and compliance during anesthesia induction (Induction Compliance Checklist). Secondary outcomes included preoperative fear (Children's Fear Scale), procedural behavior (Procedural Behavior Rating Scale), postoperative pain (Face, Legs, Activity, Cry, Consolability scale, Wong‐Baker FACES Pain Rating Scale), postoperative delirium (Pediatric Anesthesia Emergence Delirium scale), and parental satisfaction. Data were analyzed using Review Manager 5.4.1, with results presented as weighted mean differences and odds ratios with 95% confidence intervals. Certainty of evidence (Grading of Recommendations, Assessment, Development and Evaluation) were also assessed.

**Results:**

Twelve RCTs were included. Compared to the control group, the virtual reality group had lower Modified Yale Preoperative Anxiety Scale scores (weighted mean difference: −12.69, 95% confidence interval: −16.17 to −9.20, *p* < 0.001, low evidence), greater change in Modified Yale Preoperative Anxiety Scale scores (weighted mean difference: −9.54, 95% confidence interval: −12.98 to −6.10, *p* < 0.001, low evidence), lower Induction Compliance Checklist scores (weighted mean difference: −1.67, 95% confidence interval: −2.02 to −1.32, *p* < 0.001, low evidence), lower Children's Fear Scale scores (weighted mean difference: −2.30, 95% confidence interval: −2.54 to −2.07, *p* < 0.001, moderate evidence), lower Procedural Behavior Rating Scale scores (weighted mean difference: −1.00, 95% confidence interval: −1.12 to −0.88, *p* < 0.001, moderate evidence), lower Face, Legs, Activity, Cry, Consolability scale scores (weighted mean difference: −0.26, 95% confidence interval: −0.35 to −0.18, *p* < 0.001, moderate evidence), lower Wong‐Baker FACES Pain Rating Scale scores (weighted mean difference: −0.44, 95% confidence interval: −0.61 to −0.28, *p* < 0.001, moderate evidence), and higher parental satisfaction scores (weighted mean difference: 0.68, 95% confidence interval: 0.59 to 0.77, *p* < 0.001, moderate evidence). For categorical Induction Compliance Checklist, the virtual reality group showed significantly more perfect scores (odds ratio: 3.53, 95% confidence interval: 2.04 to 6.09, *p* < 0.001, moderate evidence) and fewer moderate and poor scores (odds ratio: 0.28, 95% confidence interval: 0.16 to 0.49, *p* < 0.001, moderate evidence). There was no statistically significant difference in emergence delirium (odds ratio: 1.05, 95% confidence interval: 0.59 to 1.89, *p* = 0.86, low evidence).

**Conclusion:**

Based on low to moderate quality evidence, preoperative virtual reality significantly improves compliance during anesthesia induction and offers possibly clinically significant improvements in preoperative anxiety, fear, procedural behavior, and parental satisfaction in pediatric patients undergoing general anesthesia. However, virtual reality shows a very low likelihood of clinical effect on postoperative pain, based on its very small effect size, and clearly no clinical effect on emergence delirium.

## 1 Introduction

Pediatric patients undergo general anesthesia (GA) for procedures ranging from routine day surgeries to complex operations [1, 2]. Despite significant advancements in anesthetic techniques improving surgical safety, perioperative challenges remain common [3]. These challenges are particularly pronounced in children, who have distinct physiological and psychological vulnerabilities, highlighting the need for tailored interventions to enhance perioperative care.

Psychological factors, notably preoperative anxiety and fear, can provoke stress responses, reduce the compliance of children, complicate anesthesia induction by increasing resistance or agitation, and delay recovery [4, 5, 6, 7]. Inadequate management of perioperative pain and fear can heighten postoperative pain by increasing psychological stress and pain sensitivity, and may also elevate the risk of delirium, particularly in children with severe anxiety or following complex surgeries [8, 9, 10, 11, 12]. Prolonged pain and delirium not only hinder short‐term recovery but may also pose long‐term cognitive risks such as memory deficits, attention problems, and reduced executive functioning [9, 10, 11, 13, 14].

Non‐pharmacological approaches, such as music therapy, play therapy, cognitive‐behavioral techniques, aromatherapy, and preoperative education, have been widely employed to reduce preoperative anxiety and fear, enhance compliance during anesthesia induction, and potentially decrease postoperative pain and delirium in pediatric patients [15, 16, 17, 18]. Among these, virtual reality (VR) has gained prominence due to its immersive and interactive capabilities. By creating an engaging virtual environment, VR helps divert attention from the surgical process to more pleasant experiences [19, 20]. Research indicates that VR effectively reduces preoperative anxiety, minimizes postoperative pain, and decreases analgesic use [21, 22, 23, 24, 25]. Compared to traditional interventions, its interactive nature enhances appeal and compliance among children, maximizing therapeutic impact [24, 25].

Recent reviews on VR in pediatric perioperative care reported inconsistent findings, with some concluding clear benefits for preoperative anxiety and fear, postoperative pain, and delirium, while others presented more limited effects or were unable to draw reliable conclusions on specific outcomes [19, 22, 24, 25, 26, 27]. Furthermore, the scope and conclusions of these prior syntheses were often constrained by several common limitations. These included small sample sizes (typically comprising only 4 to 7 included studies), a narrow focus on specific outcomes, and less strictly defined control groups, thereby hindering clear comparisons. The emergence of new randomized controlled trials (RCTs) [28, 29, 30] underscores the need for an updated meta‐analysis to clarify VR's effectiveness. This study addresses this gap by synthesizing the latest evidence from a larger body of RCTs. It employs a rigorous analytical approach, focusing on intention‐to‐treat (ITT) analysis, strictly defining control groups, and comprehensively assessing clinical significance, with the overall certainty of evidence for each outcome, to provide a comprehensive evaluation and establish evidence‐based recommendations.

This study aims to quantitatively analyze data from existing RCTs to assess the effects of VR as a preoperative intervention for pediatric patients undergoing GA. It is hypothesized that preoperative VR interventions can significantly improve various outcomes.

## 2 Methods

This meta‐analysis, conducted from December 10, 2024 to May 25, 2025, adhered to the Preferred Reporting Items for Systematic Reviews and Meta‐Analyses (PRISMA) guidelines [31] and was preregistered in the International Prospective Register of Systematic Reviews (PROSPERO) with the registration ID: CRD42024625705. Two independent reviewers (D.Z., T.T.) conducted the literature search, extracted data, assessed the methodological quality of included studies, and conducted the statistical analyses, with any discrepancies resolved through discussion with a third reviewer (S.J.).

### 2.1 Literature Search

A thorough search was conducted across PubMed, EMBASE, the Cochrane Library, and Web of Science databases, including all records available up to April 15, 2025. The search strategy used terms including (“Virtual Reality” OR “VR intervention” OR “immersive virtual reality” OR “VR therapy”) AND (“general anesthesia” OR “GA” OR “surgery under anesthesia” OR “elective surgery”) AND (“children” OR “pediatric patients” OR “child” OR “young patients” OR “adolescent”). Search queries were adapted to align with the unique requirements of each database (Table S1).

### 2.2 Inclusion and Exclusion Criteria

Inclusion criteria: (1) studies focusing on pediatric patients (< 18 years old) undergoing GA; (2) RCTs using ITT analysis; (3) studies directly comparing the use of immersive VR interventions delivered via head‐mounted displays (HMDs) with standard care without VR in preoperative preparation.

Exclusion criteria: (1) studies involving patients undergoing local anesthesia or emergency surgery; (2) studies involving pediatric patients with conditions that may impair the use or effectiveness of VR, such as severe visual or auditory impairments, significant cognitive or developmental disorders, severe psychiatric conditions, severe anxiety disorders unresponsive to non‐pharmacological interventions, or those on psychoactive medications; (3) studies where the control group received any form of VR or similar interventions; (4) studies that did not report sufficient data for meta‐analysis or did not clearly specify their use of ITT analysis; (5) non‐English studies.

### 2.3 Data Extraction

Extracted study characteristics included the first author's name, publication year, study location, sample size, patient demographics (age and gender distribution), surgical types, anesthesia protocol, and details of both VR and control group interventions.

Primary outcomes included children's anxiety immediately before anesthesia induction, assessed using the Modified Yale Preoperative Anxiety Scale (m‐YPAS) [32, 33] and Δ m‐YPAS (calculated as m‐YPAS immediately before induction minus m‐YPAS at baseline), both with an established minimal clinically important difference (MCID) of 15 [34, 35]. Compliance during induction was evaluated with the Induction Compliance Checklist (ICC) [36] as a continuous score and categorical outcomes (perfect, moderate and poor compliance). An MCID for continuous ICC is not yet established.

Secondary outcomes included preoperative fear measured by the Children's Fear Scale (CFS) [37], procedural behavior evaluated using the Procedural Behavior Rating Scale (PBRS) [38], and postoperative pain assessed with the Face, Legs, Activity, Cry, Consolability (FLACC) scale [39] and the Wong‐Baker FACES Pain Rating Scale (WBFS) [40]. For these four scales, an MCID is not yet established. Emergence delirium was defined as a Pediatric Anesthesia Emergence Delirium Scale (PAED) [41] score > 10 and analyzed as a binary outcome to compare rates. Parental satisfaction was measured on a 0 to 10 scale (0 = least satisfied, 10 = most satisfied), without an established MCID.

### 2.4 Quality Assessment

The methodological quality of the included studies was assessed using the revised Cochrane Risk of Bias Tool (RoB 2) [42] for RCTs. This tool assesses bias across five key domains: the randomization process, deviations from intended interventions, missing outcome data, outcome measurement, and selection of reported results. Each domain was rated as low risk, high risk, or some concerns, based on the criteria outlined in the RoB 2 guidelines. The overall certainty of evidence for each outcome was further assessed using the Grading of Recommendations, Assessment, Development and Evaluation (GRADE) methodology. This involved evaluating the risk of bias, inconsistency, indirectness, imprecision, and publication bias for each outcome, and then downgrading or upgrading the evidence quality based on predefined criteria. The final quality of evidence was categorized as high, moderate, low, or very low. Publication bias was assessed using funnel plots and Egger's test for outcomes with ten or more included studies, as per Cochrane Handbook recommendations.

### 2.5 Statistical Analysis

Meta‐analyses were performed using Review Manager (RevMan) version 5.4.1 (The Cochrane Collaboration, Oxford, UK). Continuous variables were analyzed as Weighted Mean Differences (WMD) with 95% Confidence Intervals (CIs), while dichotomous variables were expressed as pooled Odds Ratios (ORs) with 95% CIs. For the purpose of meta‐analysis, when continuous data were reported as median and interquartile range (IQR), these were converted to mean and standard deviation (SD) using established statistical methods in the Cochrane Handbook. Heterogeneity was evaluated using Cochrane's *Q* test and the *I* ^2^ statistic. For analyses with *I* ^2^ < 50%, a fixed‐effects model was employed, whereas a random‐effects model was adopted for *I* ^2^ ≥ 50%. For outcomes exhibiting substantial heterogeneity (*I* ^2^ ≥ 50%), sensitivity analyses were planned by successively excluding individual studies or studies with high risk of bias, and subgroup analyses were planned based on risk of bias grouping, to explore potential sources. Forest plots were used to present the pooled effect sizes, with statistical significance defined as *p* < 0.05.

## 3 Results

### 3.1 Study Selection and Characteristics

**FIGURE 1:**
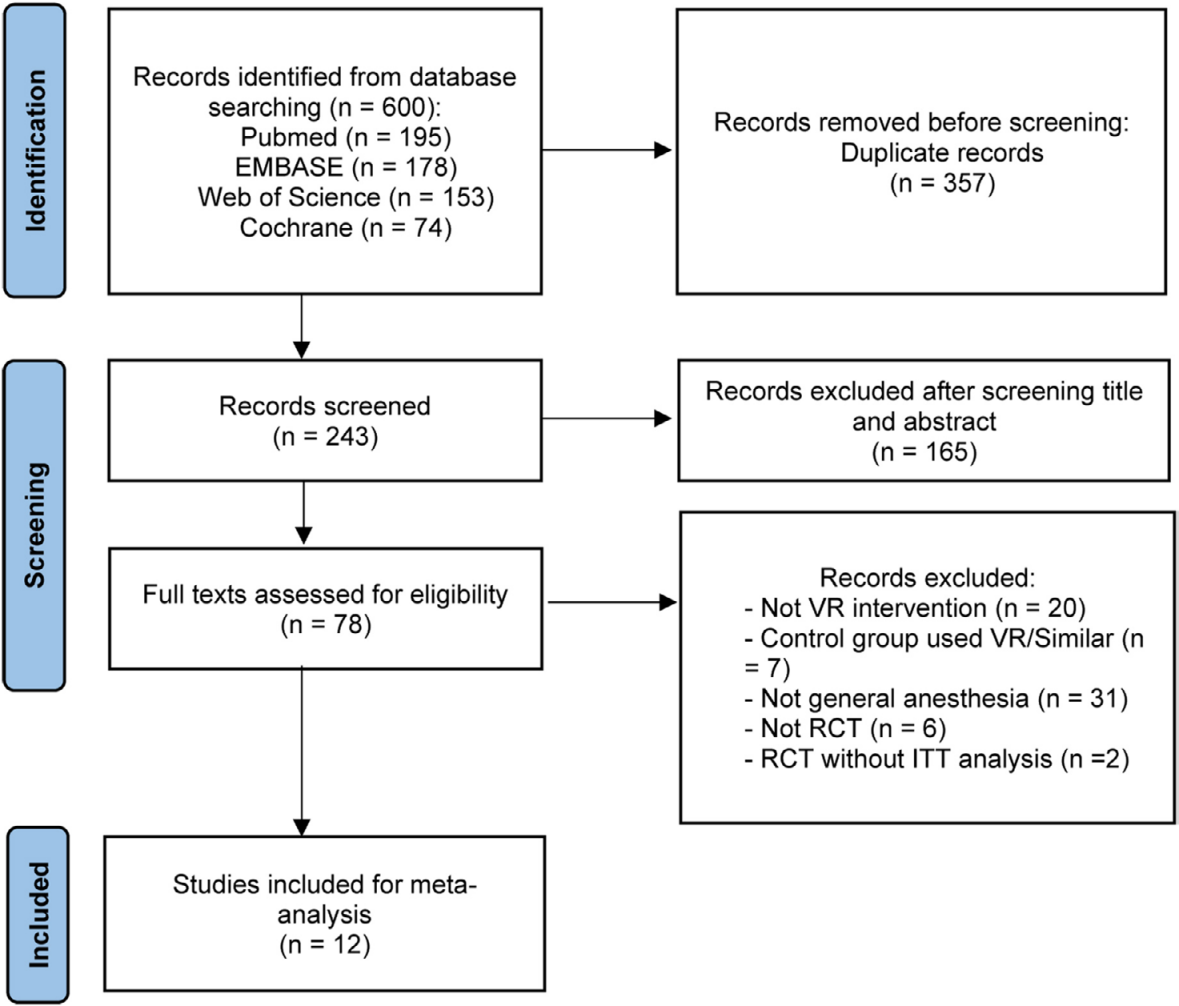
PRISMA flow chart of literature retrieval. ITT, intention‐to‐treat; RCT, randomized controlled trial; VR, virtual reality.

A search of PubMed, EMBASE, The Cochrane Library, and Web of Science found 600 studies. After removing 357 duplicates, 243 records were screened by title and abstract. Of these, 165 were excluded, leaving 78 studies for full‐text review and reference checking. After reviewing the full texts, 54 studies were excluded, resulting in 12 studies [28, 29, 30, 43, 44, 45, 46, 47, 48, 49, 50, 51] being included in the final analysis (Figure 1). The main details of these studies are presented in Table 1 and Table 2.

**TABLE 1:** Characteristics of the included studies.

First author	Year	Study location	Patients, *n*		Age, y Mean ± SD or median (IQR)		Sex (M/F)		Surgery	Anesthesia protocol
			VR	Control	VR	Control	VR	Control		
Ryu	2017	Korea	34	35	6 (5–7)	6 (5–9)	17/17	24/11	Unspecified elective surgery	IV induction (thiopental), inhaled maintenance (desflurane/sevoflurane), neuromuscular blockade (rocuronium), intubation.
Ryu	2018	Korea	34	35	5 (5–7)	6 (5–8)	18/16	22/13	Primarily otolaryngology, ophthalmology, and dental surgeries	IV/Inhalation induction (thiopental/sevoflurane), inhaled maintenance (sevoflurane), neuromuscular blockade (rocuronium), intubation.
Eijlers	2019	The Netherlands	73	97	9 (6.4–10.7)	7.5 (5.6–10.7)	34/39	56/41	Adenoidectomy, tonsillectomy, tympanostomy tube insertion, maxillofacial, and dental procedures	IV (propofol/fentanyl) or inhalation (sevoflurane) induction; sevoflurane maintenance; LMA/ETT insertion; muscle relaxant.
Ryu	2019	Korea	41	39	6 (5–7)	6 (5–8)	29/12	21/18	Primarily otolaryngology, ophthalmology, orthopedic, and dental surgeries	IV induction (thiopental, alfentanil); inhaled maintenance (sevoflurane/desflurane); neuromuscular blockade (rocuronium); intubation/LMA.
Jung	2021	USA	33	37	8.2 ± 2.2	7.8 ± 2.3	15/18	19/18	Unspecified elective surgery	Inhaled mask induction (oxygen/nitrous oxide + sevoflurane); IV catheter placement post‐induction.
Ryu	2022	Korea	34	33	5 (5–6.5)	5 (5–6)	16/19	15/20	Benign soft mass excision, inguinal hernia repair, central catheter insertion, and frenotomy	IV induction (thiopental); inhaled maintenance (sevoflurane); neuromuscular blockade (rocuronium); intubation.
Wu	2022	China	48	48	7 (6–9)	8 (5–9.8)	43/5	42/6	Circumcision, adenoidectomy and/or tonsillectomy, laparoscopic hernia repair, and local superficial mass resection	IV induction (propofol/fentanyl); LMA/ETT insertion (atracurium if intubated); sevoflurane maintenance.
Yaz	2022	Turkey	44	44	8.8 ± 2.0	8.8 ± 1.9	22/22	22/22	Unspecified elective general surgery and day surgery	Not reported.
Uysal	2023	Turkey	40	40	8.6 ± 2.0	8.7 ± 1.9	23/17	22/18	Inguinal hernia surgery	Not reported.
Carbó	2024	Spain	120	121	6.7 ± 2.9	6.2 ± 2.8	100/20	86/35	Unspecified elective surgery	Not reported.
Turgut	2024	Turkey	35	35	6.9 ± 2.1	6.8 ± 2.1	25/10	27/8	Endoscopy, hernia repair, hydrocele, hypospadias, undescended testicle, double J‐stent, cyst excision, and circumcision	Not reported.
Lee	2025	Korea	51	51	6 (5–7)	6 (5–7)	28/23	24/27	Ophthalmic, otolaryngologic, and others	Inhaled mask induction (sevoflurane/N_2_O); IV access post‐induction; IV rocuronium/alfentanil; intubation; sevoflurane maintenance.

Abbreviations: ETT, endotracheal tube; F, female; IQR, interquartile range; IV, intravenous; LMA, laryngeal mask airway; M, male; *n*, number; SD, standard deviation; VR, virtual reality.

**TABLE 2:** Characteristics of VR interventions and control groups.

First author	Year	VR device type	Content type	Content description	Duration	Timing of administration	Control group intervention
Ryu	2017	HMD (Galaxy S6 with VR Gear)	360° video	Animated tour (Pororo) covering gown change, IV placement, OR, and anesthesia induction.	4 min	1 h pre‐OR entry	Standard of care(no VR)
Ryu	2018	HMD (Oculus Rift with Leap Motion controller)	360° interactive experience	Interactive guide through preoperative process and anesthesia induction.	5 min	1 h pre‐OR entry	Standard of care (no VR)
Eijlers	2019	HMD (HTC Vive + PC monitor)	360° video	Child‐friendly OR tour with procedure explanations, age‐tailored.	~15 min	1 h pre‐OR entry	Standard of care (informative anesthesia film)
Ryu	2019	HMD (Galaxy S6 with VR Gear)	360° video	Animated tour (Pororo) covering preoperative process and anesthesia induction techniques.	4 min	1 h pre‐OR entry	Standard of care(no VR)
Jung	2021	HMD (Samsung Gear VR)	360° interactive experience	Interactive animated animal navigating a landscape.	~5 min	Immediately before GA induction in OR	Standard of care(no VR)
Ryu	2022	HMD (Oculus Go)	360° video	Tour with animated characters (Pororo) explaining preoperative process.	4 min	10 min before anesthesia	Standard of care (no VR)
Wu	2022	HMD (CV1 PRO, NOLO VR) + laptop	360° video	Story‐based video (Tantan) guiding through preoperative and anesthesia process.	5 min	1 day pre‐surgery, in separate room with parent	Standard of care (no VR)
Yaz	2022	HMD (VR goggles)	360° video	Educational animated video on preoperative and postoperative procedures.	3 min	On day of surgery, before OR entry	Standard of care(no VR)
Uysal	2023	Generic HMD (Piranha VR BOX 3.0)	360° video	Child‐selected licensed cartoons.	7–10 min	1–2 min before transfer to OR	Standard of care (no VR)
Carbó	2024	HMD (Samsung Gear VR)	360° video	Video introducing hospital areas, team, anesthesia process, age‐tailored.	5 min	7–10 days pre‐surgery, during pre‐anesthesia visit	Standard of care (no VR)
Turgut	2024	HMD (Oculus Go)	360° video	Nurse‐narrated OR tour covering preoperative and postoperative processes, age‐tailored.	1.5 min	At least 1 h pre‐procedure	Standard of care (no VR)
Lee	2025	HMD (Meta Quest 2)	360° interactive experience	Immersive 3D VR digital twin tour with interactive OR devices and animated guides.	4 min	10 min before OR entry	Standard of care (no VR)

Abbreviations: HMD, head‐mounted display; IV, intravenous; OR, operating room; PC, personal computer; VR, virtual reality.

### 3.2 Quality Assessment

**FIGURE 2:**
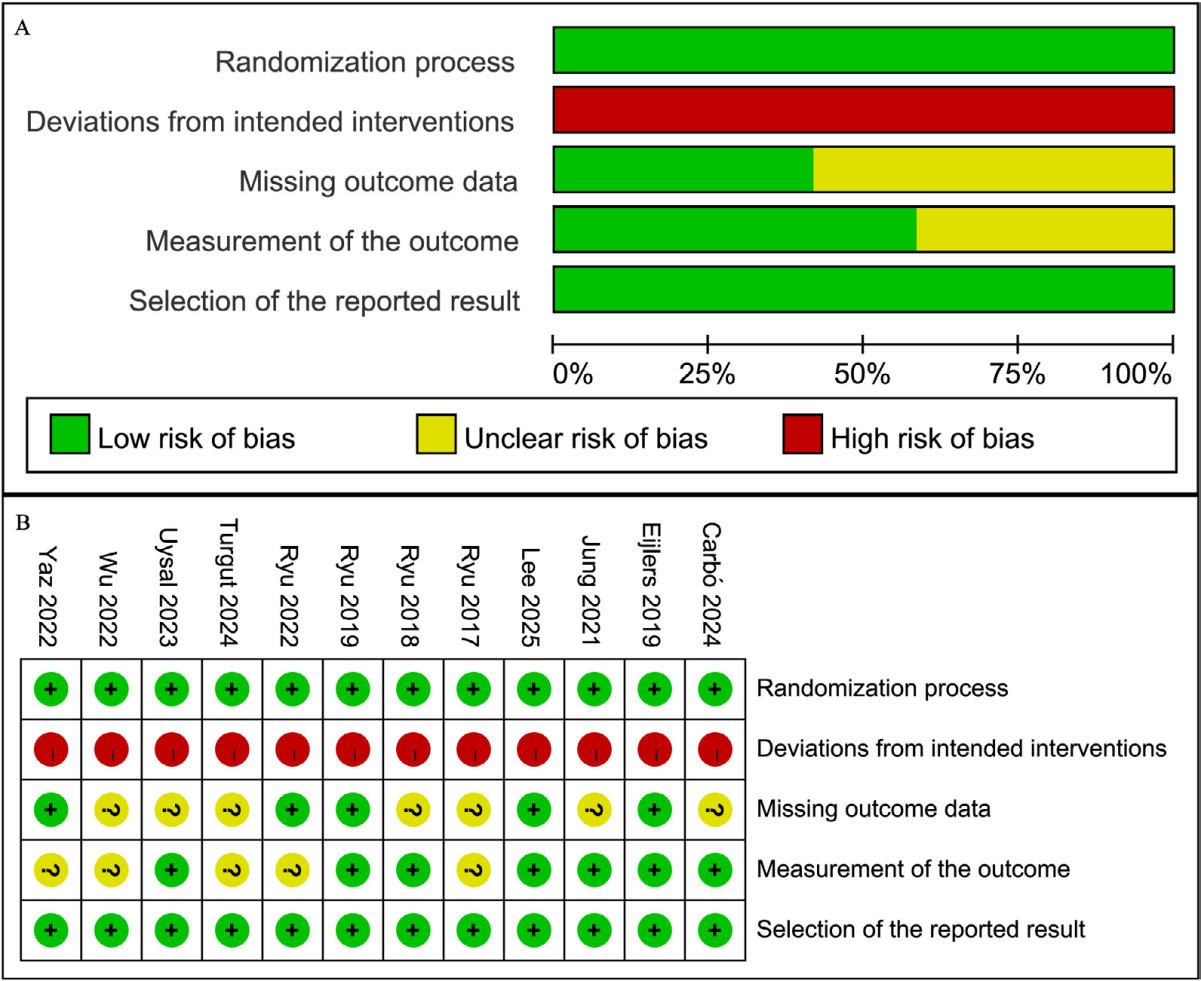
Quality assessment of the included studies.

The quality of the included studies was evaluated using the RoB 2 criteria, as illustrated in Figure 2. The primary limitation was the lack of blinding, which was unavoidable due to the nature of the intervention. As a result, all studies were rated as high risk in this domain. Additionally, some studies showed “some concerns” in the domains of missing outcome data and outcome measurement. Overall, the quality of the included studies was considered moderate.

### 3.3 Preoperative Anxiety

**FIGURE 3:**
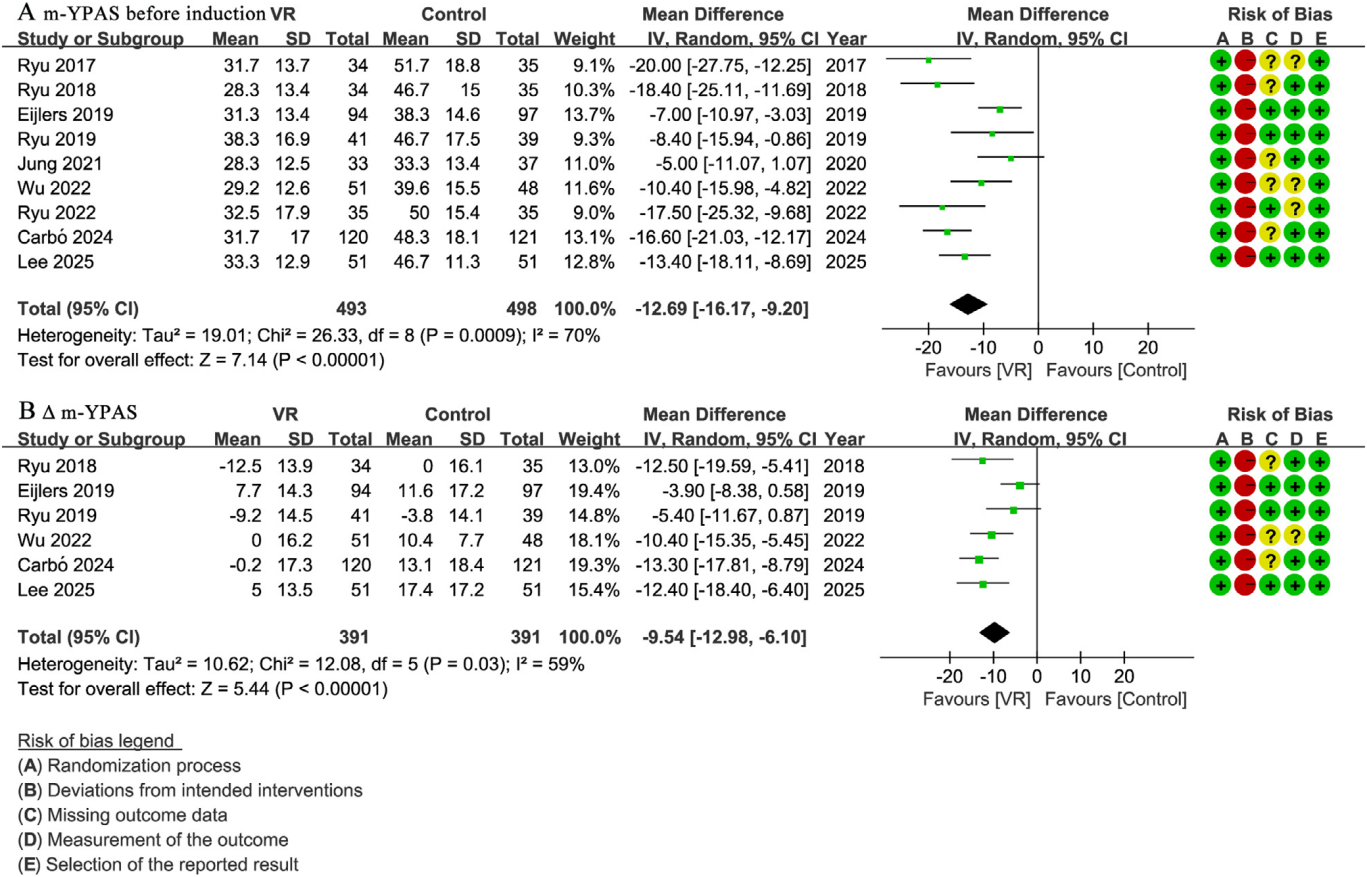
Meta‐analysis of preoperative anxiety: (A) m‐YPAS before induction, (B) Δ m‐YPAS.

Ten studies [29, 44, 45, 46, 47, 48, 49, 50, 51] involving 991 patients reported m‐YPAS before induction. The results showed that the VR group had significantly lower m‐YPAS scores compared to the control group before induction (WMD: −12.69; 95% CI: −16.17 to −9.20; *I* ^2^ = 70%; *p* < 0.001) (Figure 3A). Similarly, six studies [29, 44, 46, 48, 49, 51] with 782 patients reported Δ m‐YPAS, indicating a greater reduction in m‐YPAS in the VR group compared to the control group (WMD: −9.54; 95% CI: −12.98 to −6.10; *I* ^2^ = 59%; *p* < 0.001) (Figure 3B).

Due to the substantial heterogeneity (*I* ^2^ > 50%) observed for both m‐YPAS and Δ m‐YPAS outcomes, sensitivity analyses (by successively excluding individual studies or studies with high risk of bias) and subgroup analyses (based on risk of bias grouping) were performed. These analyses consistently showed *I* ^2^ values remaining above 50%, with the overall statistical significance and effect trends remaining unchanged. For brevity, not all individual results from these additional analyses are presented.

### 3.4 Compliance During Anesthesia Induction

**FIGURE 4:**
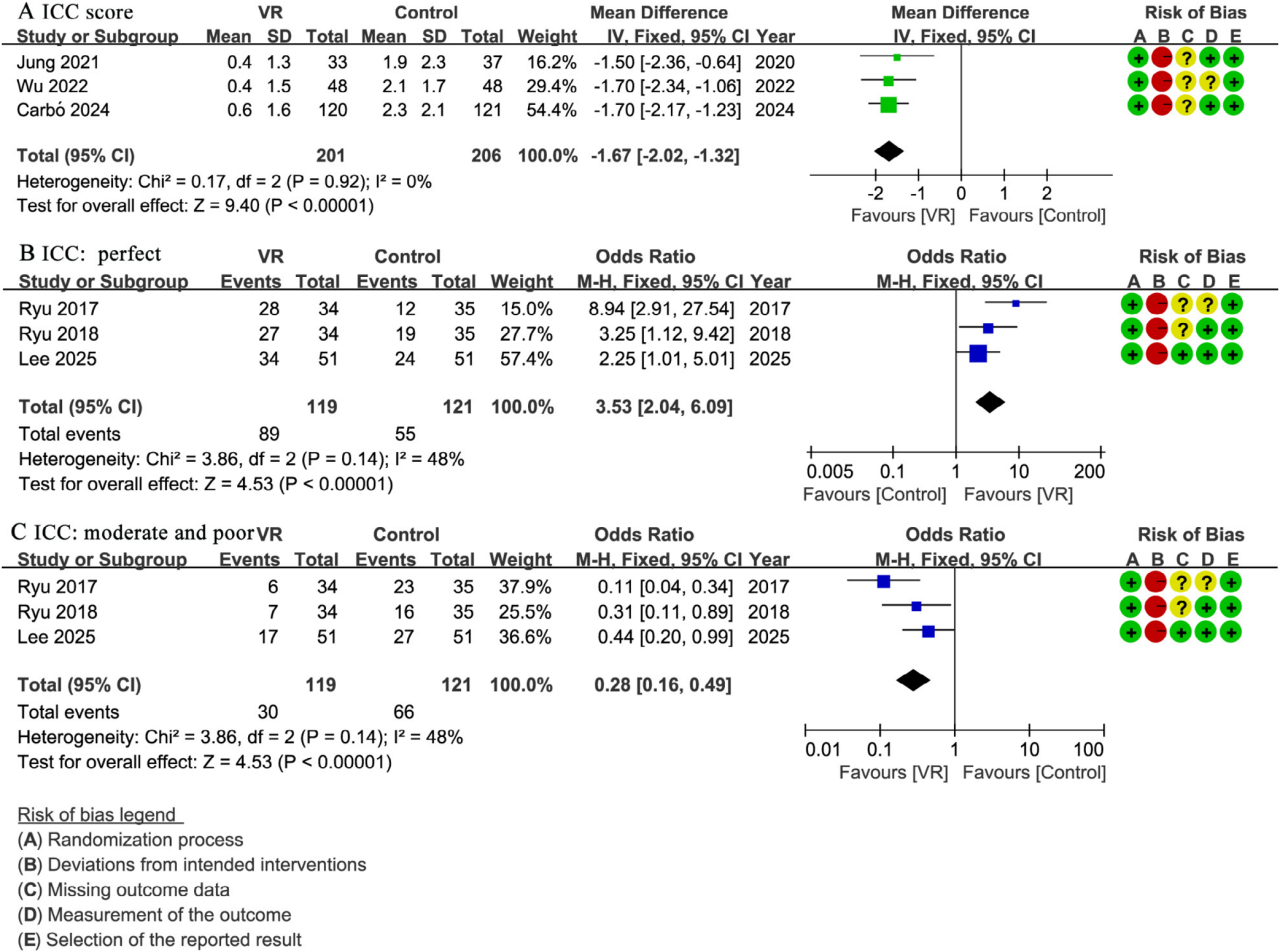
Meta‐analysis of compliance during anesthesia induction: (A) ICC, (B) ICC: 0 (perfect), (C) ICC: ≥ 1 (moderate and poor).

Three studies [29, 45, 51] involving 407 patients reported exact ICC scores. The VR group demonstrated significantly lower ICC scores compared to the control group (WMD: −1.67; 95% CI: −2.02 to −1.32; *I* ^2^ = 0%; *p* < 0.001) (Figure 4A). Three studies [46, 49, 50] with 240 patients categorized ICC outcomes as 0 (perfect), 1–3 (moderate), and ≥ 4 (poor). Compared to the control group, the VR group showed significantly more perfect scores (OR: 3.53; 95% CI: 2.04 to 6.09; *I* ^2^ = 0%; *p* < 0.001) (Figure 4B) and significantly fewer moderate and poor scores (OR: 0.28; 95% CI: 0.16 to 0.49; *I* ^2^ = 48%; *p* < 0.001) (Figure 4C).

### 3.5 Preoperative Fear and Procedural Behavior

**FIGURE 5:**
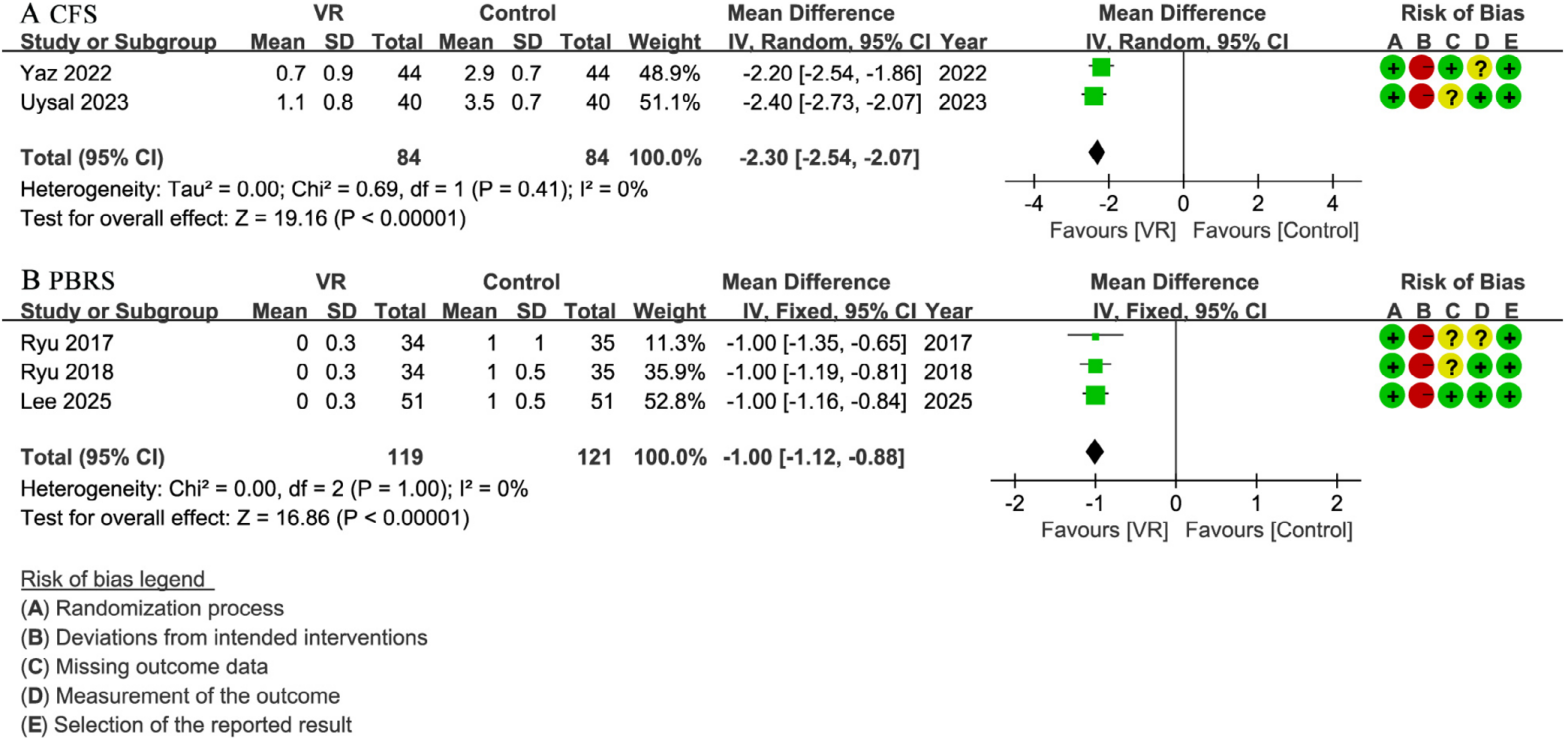
Meta‐analysis of preoperative fear and procedural behavior: (A) CFS; (B) PBRS.

Two studies [28, 43] involving 168 patients assessed fear levels using the CFS. Results showed that the VR group had statistically lower scores compared to the control group (WMD: −2.30; 95% CI: −2.54 to −2.07; *I* ^2^ = 0%; *p* < 0.001) (Figure 5A). Specifically, Uysal et al. [28] observed that children's fear in the VR group decreased after transfer to the operating room, contrasting with an increase in the control group. Similarly, Yaz et al. [43] found that a 3D VR environment designed to detail preoperative and postoperative procedures statistically reduced children's fear, while no significant change was observed in the control group.

Similarly, three studies [46, 49, 50] involving 240 patients assessed procedural behavior using the PBRS. The VR group demonstrated statistically lower PBRS scores compared to the control group (WMD: −1.00; 95% CI: −1.12 to −0.88; *I* ^2^ = 0%; *p* < 0.001) (Figure 5B). Although all three studies reported numerically lower PBRS scores in the VR group compared to the control group, the authors of these studies implied that these differences may not hold strong clinical significance in the preoperative context. They explained this might be because PBRS is more sensitive to distress experienced during painful procedures or the postoperative period, rather than the preoperative VR experience.

### 3.6 Postoperative Pain

**FIGURE 6:**
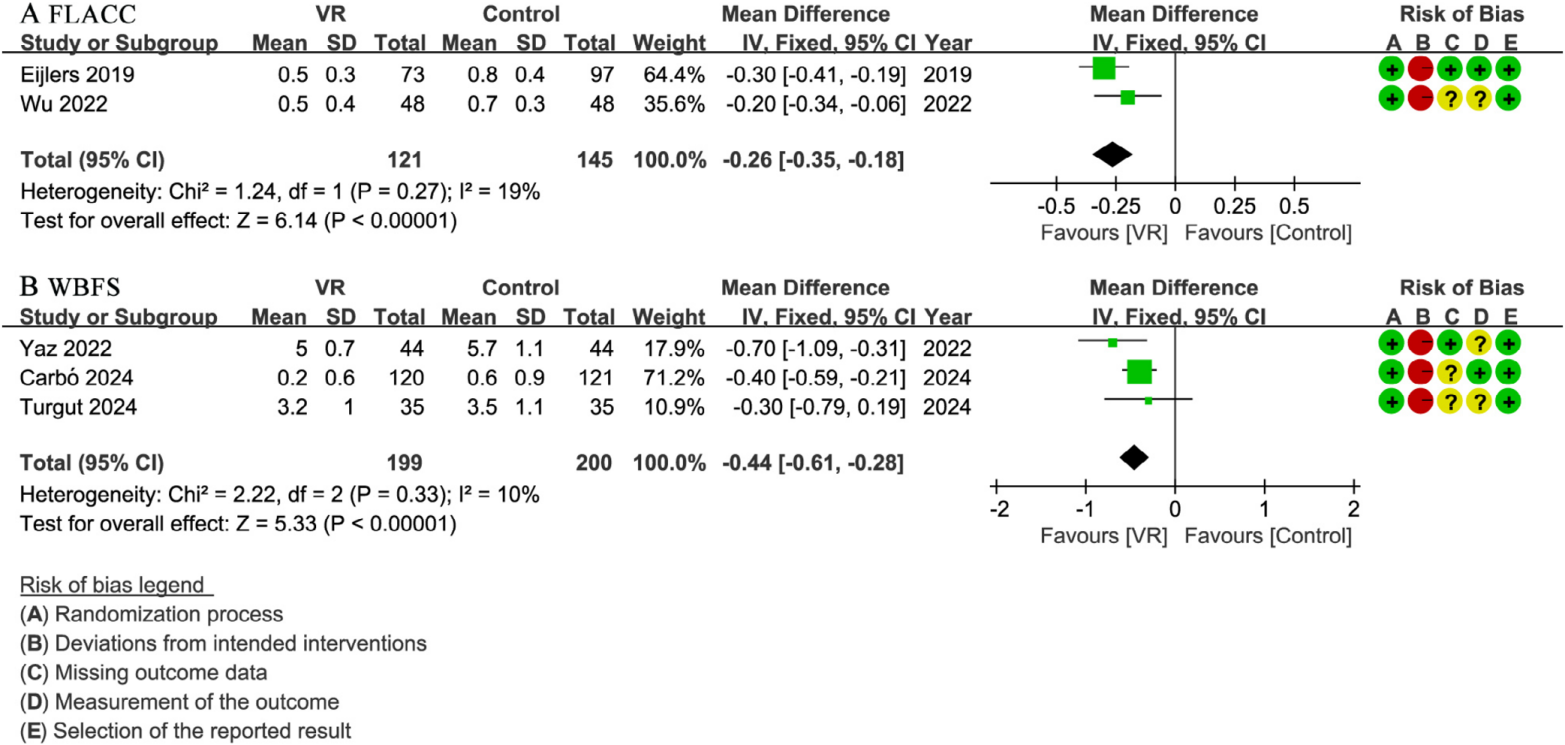
Meta‐analysis of postoperative pain: (A) FLACC, (B) WBFS.

Two studies [44, 51] involving 266 patients evaluated FLACC scores, revealing that the VR group had significantly lower scores compared to the control group (WMD: −0.26; 95% CI: −0.35 to −0.18; *I* ^2^ = 19%; *p* < 0.001) (Figure 6A). However, individually, these two studies reported very small, non‐statistically significant differences in FLACC scores. Specifically, Eijlers et al. [44] found no significant differences in nurse‐observed FLACC pain levels between groups. Similarly, Wu et al. [51] reported no statistically significant difference in FLACC scores. Despite the meta‐analysis yielding a pooled statistically significant difference, the effect's magnitude is very small (WMD: −0.26).

Similarly, three additional studies [29, 30, 43] including 399 patients assessed WBFS scores, with results favoring the VR group, which demonstrated notably lower scores (WMD: −0.44; 95% CI: −0.61 to −0.28; *I* ^2^ = 10%; *p* < 0.001) (Figure 6B). Yaz et al. [43] and Carbó et al. [29] both found statistically lower WBFS scores in the VR group compared to the control group. Conversely, Turgut et al. [30] found no statistically significant difference in WBFS scores between the two groups. Despite the meta‐analysis showing a pooled statistically significant difference, the magnitude of this effect is very small (WMD: −0.44).

### 3.7 Postoperative Emergence Delirium

**FIGURE 7:**
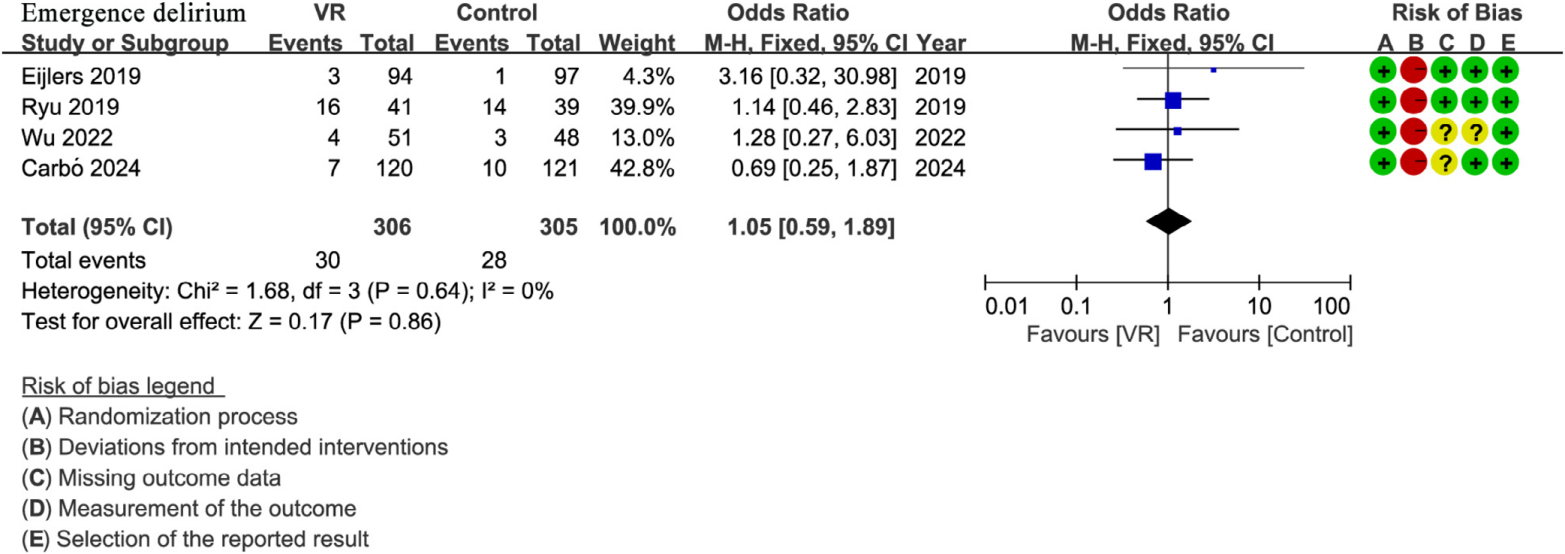
Meta‐analysis of rate of postoperative emergence delirium.

Four studies [29, 44, 48, 51] involving 611 patients assessed postoperative emergence delirium. The analysis of emergence delirium as a binary outcome (PAED score > 10) showed no statistically significant difference between the VR group and the control group (OR: 1.05; 95% CI: 0.59 to 1.89; *I* ^2^ = 0%; *p* = 0.86) (Figure 7).

### 3.8 Parental Satisfaction

**FIGURE 8:**
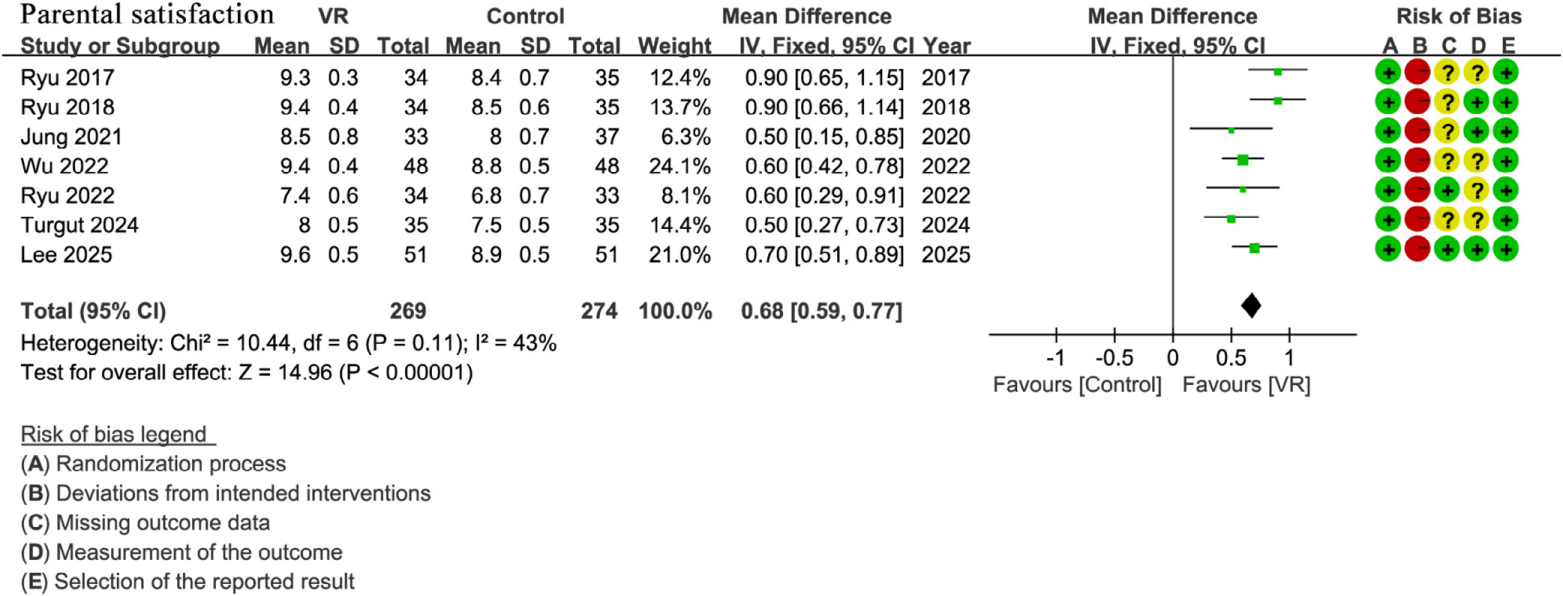
Meta‐analysis of parental satisfaction.

Seven studies [30, 45, 46, 47, 49, 50, 51] with a total of 543 patients assessed parental satisfaction scores. The results showed that the VR group had statistically higher satisfaction scores compared to the control group (WMD: 0.68; 95% CI: 0.59 to 0.77; *I* ^2^ = 43%; *p* < 0.00001) (Figure 8).

### 3.9 Quality of Evidence

The GRADE methodology was applied to assess the certainty of evidence for each outcome (Table 3). The quality of evidence was low for m‐YPAS before induction, Δ m‐YPAS, ICC score, and emergence delirium, and moderate for ICC (perfect), ICC (moderate and poor), CFS, PBRS, FLACC, WBFS, and parental satisfaction.

**TABLE 3:** GRADE summary of findings.

Preoperative VR for pediatric general anesthesia					
Patient or population: Pediatric patients undergoing general anesthesia					
Intervention: Preoperative education with VR					
Comparison: Standard of care without VR					
Outcomes	Effect estimate	95% CI	No of participants (studies)	Quality of the evidence (GRADE)	Reasons for downgrading
m‐YPAS before induction	WMD: 12.69 lower in the interventional group.	16.17 lower to 9.20 lower	991 (9 studies)	⊕ ⊕ ⊝⊝ Low	1. Serious risk of bias (due to lack of blinding, and some concerns in missing data/measurement domains); 2. Serious inconsistency (*I* ^2^ = 70%).
Δ m‐YPAS	WMD: 9.54 lower in the interventional group.	12.98 lower to 6.10 lower	782 (6 studies)	⊕ ⊕ ⊝⊝ Low	1. Serious risk of bias (due to lack of blinding, and some concerns in missing data/measurement domains); 2. Serious inconsistency (*I* ^2^ = 59%).
ICC score	WMD: 1.67 lower in the interventional group.	2.02 lower to 1.32 lower	407 (3 studies)	⊕ ⊕ ⊝⊝ Low	1. Serious risk of bias (due to lack of blinding, and some concerns in missing data/measurement domains); 2. Serious imprecision (due to very few studies and relatively small sample size for the outcome).
ICC: perfect	OR: 3.53 higher in the interventional group.	2.04 higher to 6.09 higher	240 (3 studies)	⊕ ⊕ ⊕⊝ Moderate	Serious risk of bias (due to lack of blinding, and some concerns in missing data/measurement domains).
ICC: moderate and poor	OR: 0.28 lower in the interventional group.	0.16 lower to 0.49 lower	240 (3 studies)	⊕ ⊕ ⊕⊝ Moderate	Serious risk of bias (due to lack of blinding, and some concerns in missing data/measurement domain).
CFS	WMD: 2.30 lower in the interventional group.	2.54 lower to 2.07 lower	168 (2 studies)	⊕ ⊕ ⊕⊝ Moderate	1. Serious risk of bias (due to lack of blinding, and some concerns in missing outcome data/measurement domains); 2. Serious imprecision (due to very few studies and small sample size for the outcome)
PBRS	WMD: 1.00 lower in the interventional group.	1.12 lower to 0.88 lower	240 (3 studies)	⊕ ⊕ ⊕⊝ Moderate	Serious risk of bias (due to lack of blinding, and some concerns in missing data/measurement domains).
FLACC	WMD: 0.26 lower in the interventional group.	0.35 lower to 0.18 lower	266 (2 studies)	⊕ ⊕ ⊕⊝ Moderate	1. Serious risk of bias (due to lack of blinding, and some concerns in missing data/measurement domains); 2. Serious imprecision (due to very few studies and small sample size for the outcome).
WBFS	WMD: 0.44 lower in the interventional group.	0.61 lower to 0.28 lower	399 (3 studies)	⊕ ⊕ ⊕⊝ Moderate	Serious risk of bias (due to lack of blinding, and some concerns in missing data/measurement domains).
Emergence delirium	OR: 1.05 higher in the interventional group.	0.59 lower to 1.89 higher	611 (4 studies)	⊕ ⊕ ⊝⊝ Low	1. Serious risk of bias (due to lack of blinding, and some concerns in missing data/measurement domains); 2. Serious imprecision (due to low number of events).
Parental satisfaction	WMD: 0.68 higher in the interventional group.	0.59 higher to 0.77 higher	242 (5 studies)	⊕ ⊕ ⊕⊝ Moderate	Serious risk of bias (due to lack of blinding, and some concerns in missing data/measurement domains).

*Note:* GRADE Working Group grades of evidence. High quality: Further research is very unlikely to change our confidence in the estimate of effect. Moderate quality: Further research is likely to have an important impact on our confidence in the estimate of effect and may change the estimate. Low quality: Further research is very likely to have an important impact on our confidence in the estimate of effect and is likely to change the estimate. Very low quality: We are very uncertain about the estimate.

Abbreviations: CFS, Children's Fear Scale; CI, confidence interval; FLACC, face, legs, activity, cry and consolability scale; GRADE, grading of recommendations, assessment, development and evaluation; ICC, induction compliance checklist; m‐YPAS, Modified Yale Preoperative Anxiety Scale; OR, Odds ratios; PBRS, procedural behavior rating scale; VR, virtual reality; WBFS, Wong‐Baker Faces Pain Rating Scale; WMD, weighted mean differences.

## 4 Discussion

To evaluate the effects of VR on pediatric patients undergoing GA, this meta‐analysis included a total of 12 RCTs. Compared to standard of care without VR, the VR group demonstrated statistically significant improvements in preoperative anxiety (m‐YPAS and Δ m‐YPAS), compliance during anesthesia induction (ICC), preoperative fear (CFS), procedural behavior (PBRS), postoperative pain (FLACC and WBFS scores), and parental satisfaction, but no statistically significant difference in the rate of postoperative emergence delirium.

For outcomes with an established MCID, the m‐YPAS (WMD: −12.69; 95% CI: −16.17 to −9.20) was borderline clinically significant, as its 95% CI crossed the MCID of 15. The Δ m‐YPAS (WMD: −9.54; 95% CI: −12.98 to −6.10) did not meet this threshold, indicating no clinical significance. For categorical ICC outcomes, the VR group showed significantly more perfect scores (OR: 3.53; 95% CI: 2.04 to 6.09) and fewer moderate and poor scores (OR: 0.28; 95% CI: 0.16 to 0.49), both indicating clinical significance. For other outcomes (ICC score, CFS, PBRS, FLACC, WBFS, and parental satisfaction) where an MCID is not yet established, the clinical relevance of the observed statistically significant differences remains to be definitively determined. Current evidence indicates that VR significantly improves compliance during anesthesia induction for pediatric patients. It also offers a possibly clinically significant improvement in preoperative anxiety and fear, procedural behavior, and parental satisfaction. However, VR shows a very low likelihood of clinical effect on postoperative pain, based on its very small effect size, and clearly no clinical effect on emergence delirium.

Some recent systematic reviews [19, 22, 24, 25] have investigated the effects of VR interventions on perioperative outcomes in pediatric patients. Alqudimat et al. analyzed seven RCTs with 832 participants, finding that VR reduced preoperative anxiety and improved anesthesia induction compliance [19]. Simonetti et al. included six RCTs with 716 patients, demonstrating VR's effectiveness in alleviating preoperative fear and anxiety [24]. Tas et al. reviewed five RCTs with 652 participants, highlighting reductions in postoperative pain and increased parental satisfaction [25]. The meta‐analysis by Chen et al., with four RCTs and 587 patients, focused on lowering postoperative delirium [22]. However, these studies shared common limitations, including small sample sizes and a narrow focus on specific outcomes, limiting their ability to comprehensively evaluate the full scope of VR's perioperative benefits.

To our knowledge, this is the most up‐to‐date meta‐analysis on this topic. Compared to these prior systematic reviews, our analysis generally aligned with their findings regarding the statistical benefits of VR, and we also concluded that VR can improve compliance during anesthesia induction. However, our conclusions diverge significantly when considering the clinical relevance of other effects. Previous studies generally suggested clear benefits in preoperative anxiety, fear, postoperative pain, and delirium. In contrast, our findings indicate that while VR statistically improves preoperative anxiety and fear, procedural behavior, and parental satisfaction, these effects are only possibly clinically significant. Furthermore, we found that while VR showed a statistically significant improvement in postoperative pain, this effect was not considered clinically meaningful. Another key difference in our analysis of delirium is that previous studies often used continuous variables to report a statistically significant reduction in postoperative delirium, whereas we analyzed emergence delirium as a binary outcome and found no statistically significant difference.

This divergence in conclusions may be attributed to several methodological differences in our meta‐analysis. A larger body of evidence was included, comprising 12 RCTs, making it a more extensive synthesis of the current literature. The analytical approach further incorporated a focus on RCTs that employed an ITT analysis. A comprehensive assessment of clinical significance was integrated, considering factors like established MCID and the proportion of change relative to the total scale range. Crucially, our study also reported the overall certainty of evidence for each outcome using the GRADE methodology, an aspect not consistently reported in prior reviews. The quality of evidence for many of our key outcomes was assessed as low to moderate, which underscores the need for cautious interpretation of the conclusions.

VR exerts both physiological [52, 53, 54, 55] and psychological [56, 57, 58] effects on pediatric patients undergoing GA. Physiologically, VR's immersive environment reduces perceived threat, likely attenuating amygdala and hypothalamic–pituitary–adrenal (HPA) axis activity, thereby lowering cortisol levels and mitigating stress responses [52, 53, 54, 55]. Psychologically, VR distracts children from stressful stimuli like medical equipment through engaging virtual content [56, 57, 58]. It also familiarizes them with the medical setting, reducing uncertainty and anticipatory fear. These combined effects foster a calmer, more cooperative preoperative state. These mechanisms directly improve compliance during anesthesia induction by diverting attention and reducing stress [56, 57], thus minimizing resistance and enhancing cooperation during this critical phase.

However, VR's effects on preoperative anxiety, procedural behavior, postoperative pain, and emergence delirium are not significantly clinically meaningful. This is likely due to the inherent low intensity and transient nature of VR's effects [58]. Brief VR sessions provide only transient distraction and stress reduction, often insufficient to achieve sustained clinical impact across these outcomes. Postoperative pain and ED are driven by complex factors [8, 9], which require prolonged intervention beyond VR's acute, preoperative application. Furthermore, practical limitations such as headset discomfort, restricted child‐friendly content, and short intervention durations may further hinder sustained engagement necessary for broader efficacy. Future VR applications could improve clinical impact in pediatric anesthesia with tailored, age‐specific content, extended exposure, and ergonomic designs.

The limitations of this study should be acknowledged. First, despite our stringent inclusion criteria, significant heterogeneity persisted in primary outcomes like m‐YPAS and Δ m‐YPAS. While sensitivity and subgroup analyses were performed, this substantial variability remained. This suggests that heterogeneity likely stems from inherent differences in VR intervention devices, specific protocols, durations, and patient populations across studies. From another perspective, this variability might also reflect a broader range of real‐world clinical scenarios, potentially enhancing the generalizability of our findings. Nevertheless, future research is crucial to explore these specific sources of heterogeneity and identify the optimal usage of VR interventions. Second, a major limitation across all included studies is the inability to blind patients and their families due to the inherent nature of VR interventions. This limitation raises concerns about potential placebo effects, which could be particularly pronounced in novel intervention settings [59]. Third, all included studies only assessed perioperative outcomes without long‐term follow‐up. Further research should explore the sustained impact of VR interventions. Fourth, although all participants underwent GA, variations in surgical types and differences in anesthesia practices and perioperative care across regions and medical centers may have influenced the results. Finally, the majority of studies relied on subjective scales for outcome assessment, lacking objective physiological measures such as electroencephalography [60] or salivary cortisol levels [61, 62]. Future studies should consider incorporating these objective indicators to enhance the robustness of findings.

## 5 Conclusion

Based on low to moderate quality evidence, preoperative VR significantly improves compliance during anesthesia induction and offers possibly clinically significant improvements in preoperative anxiety, fear, procedural behavior, and parental satisfaction in pediatric patients undergoing GA. However, VR shows a very low likelihood of clinical effect on postoperative pain, based on its very small effect size, and clearly no clinical effect on emergence delirium.

## Ethics Statement

The authors have nothing to report.

## Consent

The authors have nothing to report.

## Conflicts of Interest

The authors declare no conflicts of interest.

## Supporting information


Table S1.

## Data Availability

The data that support the findings of this study are available on request from the corresponding author. The data are not publicly available due to privacy or ethical restrictions.
