# Supplementary material for: Preoperative Virtual Reality for Pediatric Patients Undergoing General Anesthesia: A Meta‐Analysis of Randomized Controlled Trial
Source: Paediatr Anaesth. 2025 Jul 23;35(11):889–903. doi: 10.1111/pan.70016 (PMC12501672; doi:10.1111/pan.70016)
Supplement: Supplementary file 1 — Table S1. [file PAN-35-889-s001.docx]

**Supplementary Table 1** Search terms for each database

| Database | Search terms |
| --- | --- |
| PubMed | ("Virtual Reality"[MeSH Terms] OR "Virtual Reality" OR "VR intervention" OR "immersive virtual reality" OR "VR therapy") AND ("Anesthesia, General"[MeSH Terms] OR "general anesthesia" OR "GA" OR "surgery under anesthesia" OR "elective surgery") AND ("Child"[MeSH Terms] OR "Pediatrics"[MeSH Terms] OR "children" OR "pediatric patients" OR "child" OR "young patients" OR "adolescent"[MeSH Terms] OR "adolescent") |
| EMBASE | ('virtual reality'/exp OR 'virtual reality' OR 'VR intervention' OR 'immersive virtual reality' OR 'VR therapy') AND ('general anesthesia'/exp OR 'general anesthesia' OR 'GA' OR 'surgery under anesthesia' OR 'elective surgery') AND ('child'/exp OR 'pediatric patient'/exp OR 'children' OR 'pediatric patients' OR 'child' OR 'young patients' OR 'adolescent'/exp OR 'adolescent') |
| Cochrane Library | ("Virtual Reality" OR "VR intervention" OR "immersive virtual reality" OR "VR therapy") AND ("general anesthesia" OR "GA" OR "surgery under anesthesia" OR "elective surgery") AND ("children" OR "pediatric patients" OR "child" OR "young patients" OR "adolescent") in All Text |
| Web of Science | ("Virtual Reality" OR "VR intervention" OR "immersive virtual reality" OR "VR therapy") AND ("general anesthesia" OR "GA" OR "surgery under anesthesia" OR "elective surgery") AND ("children" OR "pediatric patients" OR "child" OR "young patients" OR "adolescent") |
